# The Construction and Evaluation of Reference Spectra for the Identification of Human Pathogenic Microorganisms by MALDI-TOF MS

**DOI:** 10.1371/journal.pone.0106312

**Published:** 2014-09-02

**Authors:** Di Xiao, Changyun Ye, Huifang Zhang, Biao Kan, Jingxing Lu, Jianguo Xu, Xiugao Jiang, Fei Zhao, Yuanhai You, Xiaomei Yan, Duochun Wang, Yuan Hu, Maojun Zhang, Jianzhong Zhang

**Affiliations:** 1 State Key Laboratory for Infectious Disease Prevention and Control, National Institute for Communicable Disease Control and Prevention, Chinese Center for Disease Control and Prevention, Beijing, China; 2 Collaborative Innovation Center for Diagnosis and Treatment of Infectious Diseases, Hangzhou, China; Harvard Medical School, United States of America

## Abstract

Matrix-assisted laser desorption/ionization time-of-flight mass spectrometry (MALDI-TOF MS) is an emerging technique for the rapid and high-throughput identification of microorganisms. There remains a dearth of studies in which a large number of pathogenic microorganisms from a particular country or region are utilized for systematic analyses. In this study, peptide mass reference spectra (PMRS) were constructed and evaluated from numerous human pathogens (a total of 1019 strains from 94 species), including enteric (46 species), respiratory (21 species), zoonotic (17 species), and nosocomial pathogens (10 species), using a MALDI-TOF MS Biotyper system (MBS). The PMRS for 380 strains of 52 species were new contributions to the original reference database (ORD). Compared with the ORD, the new reference database (NRD) allowed for 28.2% (from 71.5% to 99.7%) and 42.3% (from 51.3% to 93.6%) improvements in identification at the genus and species levels, respectively. Misidentification rates were 91.7% and 57.1% lower with the NRD than with the ORD for genus and species identification, respectively. Eight genera and 25 species were misidentified. For genera and species that are challenging to accurately identify, identification results must be manually determined and adjusted in accordance with the database parameters. Through augmentation, the MBS demonstrated a high identification accuracy and specificity for human pathogenic microorganisms. This study sought to provide theoretical guidance for using PMRS databases in various fields, such as clinical diagnosis and treatment, disease control, quality assurance, and food safety inspection.

## Introduction

The rapid and accurate identification of pathogens plays an important role in public health-related fields, such as the clinical diagnosis of infections, the prevention and control of infectious diseases, and food safety inspection. An ideal diagnostic method for infectious diseases should not only be fast, reliable, and safe but should also generate easily interpreted results at a reasonable cost. Existing pathogen diagnostic methods include immunological approaches, molecular diagnostic techniques, and identification through phenotypic characteristics and/or biochemical reactions. Although these different diagnostic methods offer various advantages, none of these approaches are capable of ideally satisfying all pathogen diagnostic requirements or are applicable for high-throughput pathogen diagnosis. Therefore, the exploration of new diagnostic methods has continued to remain a focus of research efforts. Matrix-assisted laser desorption/ionization time-of-flight mass spectrometry (MALDI-TOF MS) is an emerging technique for the rapid and high-throughput identification of microorganisms. Typically, a new approach must undergo long-term and thorough verification before it is accepted for widespread global use. At present, scientists around the world continue to evaluate and improve the capabilities of MALDI-TOF MS for the identification of various samples, including clinical isolates [1]–[3], yeasts, fungi [4]–[6], and aerobic, microaerophilic, and anaerobic microorganisms [7]–[9] Researchers are also striving to accurately assess the capabilities of the MALDI-TOF MS approach for the identification of specific pathogenic genera and species [10]–[18]. However, there remains a dearth of studies in which numerous pathogenic microorganisms from a particular country or region are utilized for systematic analyses of the MALDI-TOF MS system. These analyses are essential for the detailed evaluation of this system.

In this study, peptide mass reference spectra (PMRS) were constructed from numerous human pathogens, including enteric, respiratory, zoonotic, and nosocomial pathogens, that were selected based on infectious disease classifications and the importance, reported risks, and potential risks posed by each pathogen. These PMRS were used to enhance an existing database that had previously included spectra for numerous Western strains. Furthermore, in this investigation, systematic analyses of the enhanced database were conducted to evaluate the accuracy and specificity of pathogen identification. This study provides theoretical evidence for the applicability of the MALDI-TOF MS approach in various fields, such as clinical practice, the prevention and control of infectious diseases, quality assurance, and food safety inspection.

## Materials and Methods

### The selection and confirmation of pathogens

In this study, a total of 1019 strains of enteric, respiratory, zoonotic, and nosocomial pathogens were selected based on infectious disease classifications and the importance, reported risks, and potential risks posed by the infectious diseases associated with these pathogens. These 1019 strains included 74 international standard strains and 945 (92.7%) isolated strains. In particular, 921 (90.3%) of the selected strains were Chinese isolates obtained in different years from various geographical locations. All strain identities were confirmed by molecular and biochemical analyses, and strains were cultured in accordance with appropriate standard culture methods.

### Sample preparation and data acquisition

Samples of the examined strains were pre-extracted using previously described procedures [2]. Samples from liquid *Mycoplasma*, *Leptospira*, and *Borrelia burgdorferi* cultures were subjected to the following treatment prior to protein extraction. Cultures were collected and centrifuged at 12,000×*g* at 4°C for 10 min, and the resulting supernatants were discarded. Cell pellets were resuspended in sterile physiological saline and then centrifuged at 12,000×*g* at 4°C for 10 min; the resulting supernatants were again discarded. A Microflex LT (Bruker Daltonics, Bremen, Germany) MALDI-TOF MS system was used in this study, and the database of PMRS consulted in this investigation was provided by the Biotyper 3.0 software package (Bruker Daltonics, Bremen, Germany). MALDI-TOF MS Biotyper system (MBS) parameters were established and sample acquiring methods were performed in accordance with previously described procedures [2]. Twenty spots were dropped onto a sample target for acquiring twenty spectra for a reference spectrum construction. Two spots were prepared for each strains for validation. The highest peak intensity of each spectrum was more than 10,000.

### The construction of PMRS

PMRS were constructed as previously described [19]. The parameters used were as follows: Desired mass error for main spectra projection (MSP), 200; desired peak frequency minimum, 25%; and max. desired peak number for the MSP: 70. For each database entry, 20 individually measured mass spectra were imported into the MSP.

### The evaluation of PMRS

A cross-validation method was utilized in which database searches were performed that excluded reference spectra for the strain that was to be identified. Species containing only one strain were not validated. Identification was determined using Biotyper system parameters in which all scores ranged from 0 to 3, and identifications with scores higher than 1.7, 2.0, and 2.3 were regarded as genus identifications, species identifications, and species identifications at a high confidence level, respectively. Identification performances were classified into the following three categories: a lack of identification capability, misidentification, and accurate identification. In this study, these categories were used for database analyses to evaluate the identification accuracy and specificity (the misidentification rate) generated using the original reference database (ORD) and the new reference database (NRD) of the Biotyper system at both the genus and species levels.

### Statistical analysis

The SPSS 19.0 software package was used to analyze experimental data. The same set of spectra was used in queries of the two different databases (ORD and NRD) examined in this experiment, and statistical analyses were performed by utilizing the resulting scores as the two variables for paired t-tests. P<0.05 was regarded as a significant difference.

## Results

### The construction of the PMRS and supplementation of the ORD

In this study, PMRS were constructed from 1019 pathogen strains (886 bacterial strains, 64 *Mycoplasma* strains, and 69 spirochaetal strains) from 94 species (83 bacterial species, 6 *Mycoplasma* spp., and two *Spirochaeta* spp.) in 31 genera. The examined strains included 479 strains from 46 species of enteric pathogens, 211 strains from 21 species of respiratory pathogens, 152 strains from 17 species of zoonotic pathogens, and 177 strains from 10 species of nosocomial pathogens (The raw peak list information of the strains (exclude the high pathogenic microorganisms) used in this study were supplied as File S1). The ORD was enhanced by the addition of not only novel PMRS for 23 species in four genera (*Brucella, Leptospira, Bartonella*, and *Mycoplasma*) but also supplementary PMRS for 380 strains from 52 species, which were used to address deficiencies in database content (Table 1).

**Table 1:** Evaluation of the different reference databases.

Syndromes	Genus	Strains number (genus)	Species	PMRS number constructed	PMRS number in ORD	Identification searching ORD (genus/Species)	Misidentification by searching ORD (genus/species)	Identification searching NRD (genus/species)	Misidentification by searching NRD (genus/species)
**Enteric pathogens**	Bacillus	6	cereus	6	3	6/6	0/0	6/6	0/0
	Proteus	16	mirabilis	16	9	16/16	0/0	16/16	0/0
	Serratia	10	marcescens	10	8	10/10	0/0	10/10	0/0
	Campylobacter	24	jejuni	24	11	23/22	0/0	24/24	0/0
	Citrobacter	10	freundii	10	4	10/10	0/0	10/10	0/0
	Helicobacter	56	pylori	56	7	9/3	0/0	55/52	0/0
	Cronobacter	19	dublinensis	5	0	4/0	1/1	5/5	0/0
			malonaticus	6	0	6/0	0/4	6/5	0/1
			muytiensii	1	0	1/0	0/1		
			sakazakii	6	5	6/4	0/0	6/6	0/0
			turicensis	1	0	1/0	0/0		
	Enterobacter	20	aerogenes	9	3	9/9	0/0	9/9	0/0
			cloacae	11	16	11/7	0/2	11/10	0/1
	Escherichia	82	Ecoli	82	11	82/69	0/0	82/82	0/0
	Enterococcus	20	faecium	10	9	10/10	0/0	10/10	0/0
			faecalis	10	8	10/10	0/0	10/10	0/0
	Salmonella	102	wandsworth	2	0	2/0	0/2	2/1	0/1
			typhi	17	0	15/0	0/10	17/17	0/0
			weltevreden	2	0	2/0	0/2	2/2	0/0
			Virchow	2	0	2/0	0/2	2/0	0/2
			Uganda	2	0	2/0	0/2	2/1	0/1
			Thompson	18	0	18/0	0/18	18/12	0/6
			Stanley	2	1	2/1	0/1	2/2	0/0
			Singapore	2	0	2/0	0/2	2/0	0/2
			senftenberg	12	0	12/0	0/12	12/10	0/2
			Newport	2	0	2/0	0/2	2/0	0/2
			meleagridis	2	0	2/0	0/2	2/0	0/2
			livingstone	2	0	2/0	0/2	2/1	0/1
			Kentucky	2	0	2/0	0/2	2/1	0/1
			Istanbul	2	0	2/0	0/2	2/2	0/0
			derby	8	0	8/0	0/8	8/4	0/4
			agona	5	0	5/0	0/5	5/1	0/4
			Aberdeen	2	0	2/0	0/2	2/1	0/1
			paratyphi A	18	0	18/0	0/14	18/18	0/0
	Vibrio	57	cholerae	20	0	6/0	0/1	20/19	0/0
			fluvialis	19	3	18/11	0/0	19/19	0/0
			mimicus	5	1	3/0	0/1	5/5	0/0
			parahaemolyticus	13	7	12/10	0/0	13/13	0/0
	Listeria	20	monocytogenes	20	5	20/20	0/0	20/20	0/0
	Plesiomonas	11	shigelloides	11	5	11/11	0/0	11/11	0/0
	Aeromonas	15	caviae	5	4	5/3	0/2	5/5	0/0
			hydrophilia	10	4	10/4	0/6	10/9	0/1
	Shigella	11	boydii	2	0	0/0	2/2	2/2	0/0
			dysenteriae	2	0	0/0	2/2	2/0	0/2
			flexneri	3	0	0/0	3/3	3/3	0/0
			sonnei	4	0	0/0	4/4	3/2	1/1
**Respiratory pathogens**	Bordetella	4	bronchiseptica	1	8	1/1	0/0		
			parapertussis	1	11	1/1	0/0		
			pertussis	2	9	2/2	0/0	2/2	0/0
	Haemophilus	25	haemolytius	15	0	10/0	0/0	15/15	0/0
			influenzae	10	10	10/10	0/0	10/10	0/0
	Klebsiella	16	pneumoniae	16	122	16/16	0/0	16/16	0/0
	Legionella	20	bozemanii	1	4	0/0	0/0		
			dumoffii	1	6	1/1	0/0		
			fairfieldensis	1	0	0/0	0/0		
			micdadei	1	1	1/1	0/0		
			penumophila	16	10	16/15	0/0	16/16	0/0
	Mycoplasma	64	fermentans	1	0	0/0	0/0		
			genitalium	1	0	0/0	0/0		
			hominis	1	0	0/0	0/0		
			penetrans	1	0	0/0	0/0		
			pirum	1	0	0/0	0/0		
			pneumoniae	59	0	0/0	0/0	59/59	0/0
	Streptococcus	64	pneumoniae	20	9	20/20	0/0	20/20	0/0
			pyogenes	24	8	24/24	0/0	24/24	0/0
			suis	20	3	20/20	0/0	20/20	0/0
	Neisseia	18	meningitidis	18	11	18/18	0/0	18/18	0/0
**Zoonotic pathogens**	Bartonella	10	bacilliformis	1	0	0/0	0/0		
			clarridgeiae	1	0	0/0	0/0		
			elizabethae	1	0	0/0	0/0		
			grahamii	1	0	0/0	0/0		
			henselae	1	0	0/0	0/0		
			quintana	1	0	0/0	0/0		
			tribocorum	1	0	0/0	0/0		
			vinsonii	3	0	0/0	0/0	3/3	0/0
	Borrelia	4	burgdorferi	4	1	1/0	0/0	4/3	0/0
	Brucella	61	abortus	15	0	0/0	0/0	15/10	0/5
			canis	3	0	0/0	0/0	3/2	0/1
			neotomae	1	0	0/0	0/0		
			suis	5	0	0/0	0/0	5/1	0/4
			melitensis	37	0	0/0	0/0	37/33	0/4*
	Burkholderia	11	pseudomallei	11	0	10/0	0/4	11/10	0/1
	Leptospira	65	biflexa	22	0	0/0	0//0	22/22	0/0
			interrogans	43	0	0/0	0/0	43/43	0/0
**Nosocomial pathogens**	Staphylococcus	122	aureus	122	12	122/122	0/0	122/122	0/0
	Clostridium	10	difficile	10	10	10/10	0/0	10/10	0/0
	Acinetobacter	45	baumannii	20	7	17/9	0/0	20/19	0/0
			bereziniae	3	0	3/0	0/3	3/3	0/0
			haemolyticus	1	2	1/1	0/0		
			johnsonii	1	5	1/1	0/0		
			junii	6	2	6/6	0/0	6/6	0/0
			lwoffii	1	7	1/1	0/0		
			Unosocomialis	5	0	2/0	0/1	5/3	0/2
			pittii	8	18	7/2	0/0	8/4	0/2
**Total**	31	1019	94	1019*		720*/517*	12*/126*		1*/54*
				995^#^		711^#^/510^#^	12^#^/126^#^	992^#^/931^#^	1^#^/54^#^
				631@		572@/510@	0@/12@	630@/620@	0@/4@

*indicates statistical analyses for all strains that were newly incorporated into the database; # indicates the strains excluding single-strain species; @ indicates the strains with reference spectra in the ORD. ORD: The original reference spectra of Biotyper 2.0 (3995). NRD: The original reference spectra of Biotyper 2.0 with the 1019 reference spectra constructed herein (5014).

### Evaluations of MBS identification accuracy

**Figure 1:**
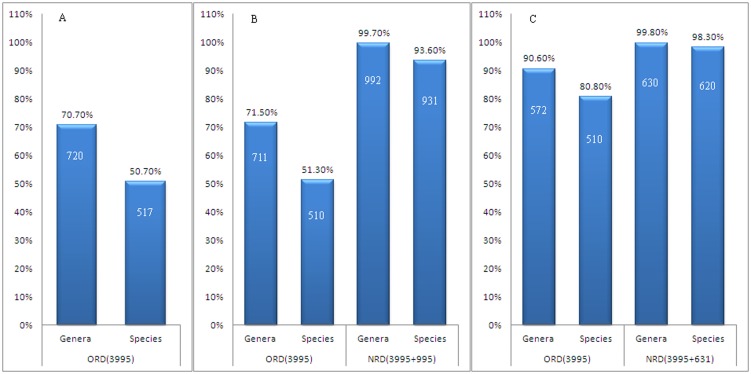
Comparisons of the identification accuracies generated using the ORD and the NRD for pathogenic microorganisms. A: The identification of 1019 strains using the ORD; B: The identification of pathogens (after excluding 24 strains from single-strain species) using the ORD and the NRD; C: The identification of pathogens (after excluding 24 strains from single-strain species and strains without reference spectra in the ORD) using the ORD and the NRD.

Of the 1019 pathogen strains used in this study, 720 strains (70.7%) and 517 strains (50.7%) were accurately identified at the genus and species levels, respectively, in searches of the ORD. Cross-validation analyses indicated that of the 995 strains remaining after the exclusion of the 24 strains that were the only examined strain for a species, 711 strains (71.5%) and 510 strains (51.3%) were accurately identified at the genus and species levels, respectively, in searches of the ORD (which included 3995 PMRS). In contrast, 992 strains (99.7%) and 931 strains (93.6%) were accurately identified at the genus and species levels, respectively, in searches of the NRD (which included 5014 PMRS). To exclude differences in identification accuracy produced by a lack of appropriate PMRS at the species level, analyses were performed using only pathogens with PMRS in the ORD. In these analyses of 631 strains, 572 strains (90.6%) and 510 strains (80.8%) were accurately identified at the genus and species levels, respectively, in searches of the ORD, whereas 630 strains (99.8%) and 620 strains (98.3%) were accurately identified at the genus and species levels, respectively, in searches of the NRD (Figure 1).

### Analyses of MBS identification specificity

**Figure 2:**
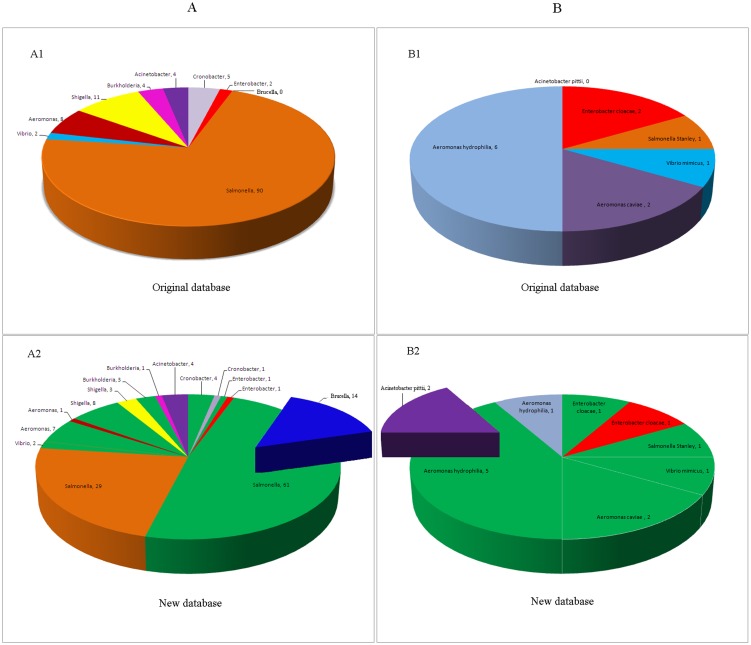
A statistical chart indicating misidentification rates before and after database expansion. A: Distributions of pathogen misidentifications (after excluding 24 strains from single-strain species) generated using the ORD and the NRD; A1: The distribution of pathogen misidentifications generated using the ORD for 995 strains; A2: The distribution of pathogen misidentifications generated using the NRD for 995 strains; B: Distributions of pathogen misidentifications (after excluding 24 strains from single-strain species and strains without reference spectra in the ORD) generated using the ORD and the NRD; B1: The distribution of pathogen misidentifications generated using the ORD for 631 strains; B2: The distribution of pathogen misidentifications generated using the NRD for 631 pathogenic strains. Green indicates the strains that were misidentified using the ORD but accurately identified by searching the NRD. Bold text indicates strains that were misidentified by the NRD.

Of the 995 examined pathogen strains, 12 strains (1.2%) and 126 strains (12.7%) were misidentified at the genus and species levels, respectively, in searches of the ORD. At the genus level, 11 *Shigella* strains were misidentified as *Escherichia* strains, and one *Cronobacter* strain was misidentified as an *Enterobacter* strain; at the species level, major misidentifications included 31 species from 8 genera, including *Salmonella*, *Shigella*, *Cronobacter*, *Enterobacter*, *Vibrio*, *Aeromonas*, *Burkholderia*, and *Acinetobacter* (Table 1, Figure 2 A1). In searches of the NRD, one strain was misidentified at the genus level (0.1%); one *Shigella* strain was misidentified as an *Escherichia coli*. At the species level, 54 strains (5.4%) were misidentified; specifically, *Vibrio cholera* and *Vibrio mimicus* strains were easily misidentified as strains of the closely related species *Vibrio albensis*, poor species identification was observed for *Cronobacter* spp., and *Enterobacter cloacae* and *Enterobacter asburiae* were indistinguishable. In addition, suboptimal species specificity was observed for various *Salmonella* serotypes, four *Shigella* serogroups, and *Brucella* species. In total, species-level misidentifications involved pathogenic strains from 8 genera and 25 species (Table 1, Figure 2 A2).

The 631 examined strains with PMRS in the ORD were all correctly identified at the genus level in searches of either the ORD or the NRD. At the species level, searches of the ORD led to the misidentification of 12 strains (1.9%), including *Enterobacter cloacae*, *Salmonella* Stanley, *Vibrio mimicus*, *Aeromonas caviae*, and *Aeromonas hydrophila* (Table 1, Figure 2 B1), whereas searches of the NRD led to the misidentification of four strains (0.6%), including *Enterobacter cloacae*, *Aeromonas hydrophila*, and *Acinetobacter pittii* (Table 1, Figure 2 B2).

## Discussion

Studies by other researchers and our preliminary investigations have demonstrated that database quality is a key factor affecting the application of an MBS for microorganism identification. A complete database of microorganism PMRS is a prerequisite for ensuring that the MBS will exhibit strong identification capabilities [11], [14], [19]–[21]. The pathogens examined in this study included 94 different pathogen species (bacterial species, *Mycoplasma* spp., and *Spirochaeta* spp.) that accounted for 60.6% of the 155 pathogenic species documented in the “Catalog of Human-borne Pathogenic Microorganisms”. This catalog, which was published by the Ministry of Health of the People's Republic of China, includes various bacterial, fungal, *Chlamydia*, *Mycoplasma*, *Rickettsia*, and *Borrelia* species. With respect to diseases, enteric pathogens (46 species, 479 strains), respiratory pathogens (21 species, 211 strains), zoonotic pathogens (17 species, 152 strains), and nosocomial pathogens (10 species, 177 strains) were included in this study. The pathogens of interest included highly pathogenic species, such as *Vibrio cholerae* and *Brucella* spp., and common foodborne pathogens (*Campylobacter jejuni*, *Staphylococcus aureus*, the enterohemorrhagic *Escherichia coli* O157:H7, *Salmonella* spp., *Shigella* spp., *Vibrio cholerae*, *Vibrio parahaemolyticus*, *Proteus* spp., and *Bacillus cereus*). The wide range of pathogen PMRS constructed in this study contributed to not only supplementing the Biotyper ORD at the genus and species levels but also adding data for numerous new pathogens to this database; thus, our results provide fundamental support for the application of the MBS in various fields, such as the clinical diagnosis of infections, the prevention and control of infectious diseases, food safety inspection, and quality assurance for export and import goods.

Previous studies reported MBS identification accuracies of 79.7–100% at the species level [11],[15]–[18],[22]–[26]. However, in this study, the accurate identification rates for 1019 strains in searches of the ORD (with 3995 PMRS) were 70.7% at the genus level and only 50.7% at the species level. The expansion and enhancement of the ORD to form the NRD (with 5014 PMRS) resulted in improvements of 28.2% (from 71.5% to 99.7%) and 42.3% (from 51.3% to 93.6%) in identification rates at the genus and species levels, respectively. This result fully validated the strength of MBS identification capabilities. Two major factors affect MBS identification accuracy. The first of these factors is the pathogen PMRS included in the database. However, the ORD could not satisfactorily identify varietal strains because of the low number and diversity of PMRS in this database. For example, *Acinetobacter pittii* and *Acinetobacter nosocomialis*, which are strains with low pathogenicity, are frequently misidentified as *Acinetobacter baumannii* in clinical diagnostics [27], leading to the adoption of inappropriate therapeutic approaches. However, in this study, a complete PMRS database enabled *Acinetobacter baumannii* to be accurately distinguished from *Acinetobacter pittii* and *Acinetobacter nosocomialis*. The second factor affecting MBS identification accuracy is that MBS databases lack PMRS for diverse high-pathogenicity strains, including various *Brucella*, *Leptospira*, and *Bartonella* strains; thus, these strains cannot be identified using the MBS. The majority of MBS users can provide only limited contributions to database improvement due to strain-related resource limitations. MBS usage demands vary by field, and pathogen identification procedures that solely rely on commercial databases cannot meet the identification needs of certain fields. The ideal solution to this issue is to develop a global platform based on a MALDI-TOF MS identification system that allows the merits of this system, such as its rapidity, accuracy, and applicability for high-throughput procedures, to be fully realized through data sharing.

High specificity is critical for pathogen identification. The consequences of pathogen misidentification are typically more harmful than the consequences of an inability to identify a pathogen because identification results greatly affect subsequent treatment and control strategies in a variety of fields, including clinical diagnosis, the prevention and control of infectious diseases, and food safety inspection. The main genus-level misidentifications produced by the MBS were the misidentification of *Shigella* and *Cronobacter* strains as *Escherichia* and *Enterobacter* strains, respectively. At the species level, the misidentification of *Salmonella* and *Brucella* species accounted for 53.7% and 25.9%, respectively, of the total misidentification events. A clear understanding of the differences in identification specificity among different pathogen peptide mass spectra will facilitate the development of scientific approaches to solve identification specificity-associated problems. For *Shigella* and *Escherichia coli*, neither the commercial Biotyper database nor the SARAMIS database contains *Shigella* PMRS; as a result, all tested *Shigella* strains would be identified as *Escherichia coli* strains, leading to misidentifications that do not improve system identification capabilities. Thankfully, we found that building the GA model using the ClinProTools software can distinguish between *Shigella* and *Escherichia coli* using peptide mass spectra (data not shown), which has also been reported recently [28]. In this study, only 8 species-level misidentifications out of the total of 54 species-level misidentifications were observed among *Salmonella* with numerous PMRS. Numerous PMRS were also available for *Brucella melitensis* and *Brucella abortus*, and these two species could be specifically identified. Overall, the use of the NRD instead of the ORD reduced misidentification rates by 91.7% and 51.7% at the genus and species levels, respectively. Thus, the database enhancements produced by this study significantly reduced misidentification rates at both the genus and species levels. The study results suggest that instead of excluding strains with low identification specificity from MBS databases, large quantities of PMRS must be constructed for these strains. Depending on the database of the user, additional tests to verify the identities of these strains may be required, and alternative identification methods should be utilized as needed.

Of the 1019 pathogen strains identified in this study, 90.3% were Chinese isolates; moreover, 91.9% of the 593 Chinese isolates with PMRS in the ORD produced spectra matching the PMRS for the Chinese isolates that were newly incorporated into the NRD. Furthermore, statistical assessments revealed significant geographical distribution patterns (Table 2), most notably *Helicobacter pylori*. After the incorporation of PMRS for Chinese isolates into the NRD, the identification rates for 56 *Helicobacter pylori* strains in this study increased from 5% to 93%. Microorganism identification using the MBS involves the examination of peptides or small protein molecules with molecular weights of 2–20 kDa; the majority of these molecules are ribosomal proteins [21],[29]. It has been demonstrated that the toxicity and pathogenicity of diverse pathogens can vary with geographical location. This geographical specificity suggests that for certain strains, ribosomal proteins might differ in various locations; as a result, these strains might exhibit varying identification specificities in different regions, and different identification potentials for these strains might be observed if the same pathogen identification system is used in different countries. The geographical specificity of peptide mass spectra of various pathogens suggests that MBS might be an ideal tool to not only classify certain pathogens but also track the source and spread of these pathogens.

**Table 2:** Statistical analyses of the scores of pathogens from the ORD and the NRD.

Species	Original database	New database	P value	T value
	Means±SD	Means±SD		
Bacillus cereus	2.29±0.053	2.625±0.139	0.000	−5.510
Proteus mirabilis	2.307±0.108	2.606±0.101	0.000	−8.09
Serratia marcescens	2.175±0.067	2.611±0.077	0.000	−13.482
Campylobacter jejuni	2.270±0.101	2.517±0.115	0.000	−7.893
Citrobacter freundii	2.363±0.122	2.587±0.137	0.001	−3.872
Helicobacter pylori	1.411±0.351	2.223±0.19	0.001	−15.207
Cronobacter sakazakii	1.982±0.175	2.544±0.115	0.000	−6.565
Enterobacter aerogenes	2.406±0.053	2.544±0.085	0.001	−7.71
Enterobacter cloacae	2.231±0.139	2.435±0.1	0.005	−3.804
Escherichia Ecoli	2.169±0.156	2.509±0.104	0.000	−25.372
Enterococcus faecium	2.331±0.075	2.749±0.035	0.000	−16.03
Enterococcus faecalis	2.338±0.101	2.673±0.097	0.000	−19.998
Vibrio fluvialis	1.97±0.177	2.451±0.114	0.000	−12.194
Vibrio mimicus	1.751±0.154	2.410±0.219	0.006	−4.54
Vibrio parahaemolyticus	2.122±0.204	2.411±0.028	0.000	−6.527
Listeria monocytogenes	2.368±0.086	2.636±0.096	0.000	−17.962
Plesiomonas shigelloides	2.074±0.048	2.607±0.014	0.000	−37.328
Aeromonas caviae	2.168±0.028	2.483±0.05	0.000	−7.844
Aeromonas hydrophilia	2.078±0.051	2.481±0.127	0.004	−5.783
Haemonphilus influenzae	2.211±0.081	2.451±0.149	0.000	−5.405
Klebsiella pneumoniae	2.414±0.119	2.539±0.117	0.006	−2.979
Legionella penumophila	2.124±0.078	2.479±0.086	0.000	−12.271
Streptococcus pneumoniae	2.331±0.105	2.513±0.102	0.000	−5.522
Streptococcus pyogenes	2.254±0.174	2.562±0.137	0.000	−6.819
Streptococcus suis	2.33±0.095	2.674±0.118	0.000	−10.179
Neisseria meningitidis	2.28±0.074	2.533±0.059	0.000	−20.23
Borrelia burgdorferi	1.431±0.367	1.999±0.164	0.085	−2.54
Staphylococcus aureus	2.565±0.067	2.859±0.055	0.000	−50.482
Clostridium difficile	2.346±0.091	2.714±0.064	0.000	−12.218
Acinetobacter baumannii	1.962±0.306	2.224±0.150	0.001	−3.428
Acinetobacter junii	2.171±0.090	2.364±0.189	0.046	−2.275
Acinetobacter pittii	1.942±0.152	2.102±0.144	0.050	−2.165

As natural and societal factors change, continued increases in the global flow of populations and materials could promote the global transmission of pathogenic microorganisms. Rapid and accurate diagnoses are vital to the battle against infectious human diseases, as these diagnoses can provide the basis for effective treatment and evidence to support the development of disease prevention strategies. MALDI-TOF MS will inevitably become the most common, accurate, high-throughput, and economical tool for the identification of the majority of pathogenic microorganisms. An ideal way to improve this identification approach is to develop a MALDI-TOF MS-based global platform that allows the merits of this system, such as its rapidity, accuracy, and applicability for high-throughput procedures, to be fully realized through data sharing.

## Supporting Information

File S1 **The original peak lists of the strains used in this study (excluding the highly pathogenic organisms.**
(ZIP)
